# The possibilities and challenges associated with selective targeting *Plasmodium falciparum* Hsp90 for Malaria

**DOI:** 10.1080/0035919x.2025.2461304

**Published:** 2025-02-07

**Authors:** Thato Matlhodi, Lisema Patrick Makatsela, Njabulo Joyfull Gumede, Fortunate Mokoena

**Affiliations:** 1 Department of Biochemistry, Faculty of Natural and Agricultural Science, North-West University, Mmabatho, South Africa; 2 Department of Chemical and Physical Sciences, Faculty of Natural Sciences, Walter Sisulu University (WSU), Private Bag X01, Umthatha, 4099, Eastern Cape, South Africa

**Keywords:** antimalarials, PfHsp90, machine learning, target-based drug discovery

## Abstract

The causative agent of malaria, *Plasmodium falciparum*, encodes four heat shock protein 90 isoforms in the cytosol, endoplasmic reticulum, mitochondria and apicoplast. PfHsp90s are considered potential targets for developing antimalarial drugs. However, the similarity between the druggable ATP binding pocket of these isoforms and their human counterparts has hindered efforts in discovering Hsp90-based antimalarial drugs. There is widespread concern that the chemotypes targeting PfHsp90 isoforms may not possess the selectivity required for translatability. Most studies have focused on the cytosolic (canonical Hsp90) and have used various inhibitors of Hsp90 to conduct anti-*Plasmodium* and mammalian safety studies without considering the on-target enzymatic activity and binding affinity. The extent to which the cytosolic Hsp90 shares common mechanisms with the other isoforms remains elusive. As such, detailed structural comparisons of the Hsp90 isoforms may reveal exploitable differences that could favour preferential binding. It is essential to consider whether molecules that act as pan-inhibitors would put more pressure on the parasite or have detrimental effects. Studies should go beyond molecular docking and whole-cell activity to advance the development of Hsp90 inhibitors as future antimalarials. There is a need also to assess the physicochemical properties, drug metabolism, pharmacokinetics, and *In vivo* proof-of-concept. We argue that the potential benefits of PfHsp90 isoform inhibitors outweigh the off-target and selectivity risks. Additionally, opportunities for using machine learning and computer-aided drug discovery efforts to design inhibitors that preferentially bind to each isoform, particularly the cytosolic PfHsp90, are highlighted as a means of overcoming the resistance challenge.

## Introduction

Malaria remains a significant health challenge, contributing to 608,000 deaths in 2022 (WHO, 2023). The disease disproportionately impacts low-income countries, especially those in sub-Saharan Africa. The region’s temperature and arid conditions create an ideal environment for the transmission of *Anopheles* mosquito vectors (Guerra et al., 2008). The World Health Organization (WHO) has identified climate change, along with rising temperatures and changing precipitation patterns, as the most critical health threat to humanity (WHO, 2023). Climate changes could affect malaria transmission and burden (Craig et al., 1999; Nissan et al., 2021; Reiter, 2001) by potentially creating more favorable environments for mosquito breeding and survival (Reiter, 2001; WHO, 2023). Moreover, climate change could expand the geographical range of malaria transmission, intensify transmission in current endemic areas, lead to the reintroduction of malaria in previously eliminated regions, or reduce transmission intensity in certain areas (Nissan et al., 2021). Therefore, addressing climate change and its health implications, including malaria, requires integrated approaches that prioritize mitigation efforts, strengthen adaptation strategies, and enhance healthcare resilience to effectively respond to emerging challenges.

Human malaria is caused by five *Plasmodium* species: *P. falciparum*, *P. vivax*, *P. malariae*, *P. knowlesi*, and *P. ovale*. Among them, *P. falciparum* is the most dangerous, causing about 95% of malaria cases worldwide (Ashley et al., 2018; Battle et al., 2019; Schofield & Grau, 2005; Weiss et al., 2019). Controlling and eliminating malaria requires a multi-pronged approach, including the use of insecticides, impregnated bed nets, vector control, vaccinations, and therapeutics (Okumu & Moore, 2011; Pryce et al., 2022). However, these interventions face challenges, such as insecticide resistance in mosquitoes. *P. falciparum* has approximately 5,500 genes and uses antigenic variation to evade the immune system. Nonetheless, the recent approval of the WHO-qualified RTS, S/AS01 vaccine represents a significant breakthrough in malaria control. A promising vaccine candidate, R21/Matrix-M, demonstrated 75% efficacy in protecting children for 12 months in specific seasonal malaria areas (Datoo et al., 2024). Notably, participants in the vaccine studies received standard measures, including insecticide-treated bed nets and seasonal malaria chemoprevention drug combinations (Datoo et al., 2024). Therefore, a promising malaria control strategy involves combining a pre-erythrocytic stage malaria vaccine with an effective chemo preventive therapy. Malaria has traditionally been controlled with chemotherapy (Greenwood et al., 2021). There is a desperate need to develop new therapies with well-defined but unexploited targets, particularly those with refractory to acquire resistance. One potential candidate is the *P. falciparum* heat shock protein 90 (Hsp90), which plays critical roles during proteostasis and has been implicated in drug resistance.

## Antimalarial drug resistance

*P. falciparum’*s resistance to antimalarial drugs has posed a persistent challenge to global malaria treatment efforts, hindering the reduction of the disease burden (Rasmussen et al., 2022). Resistance in the context of malaria refers to the capability of a parasite strain to persist and reproduce even when exposed to the correct administration and absorption of a recommended dose of antimalarial medication (Popovici et al., 2021). Over the past century and beyond, instances of resistance to malaria parasites have emerged against nearly all available antimalarial drugs at different times. Drug resistance occurs when the parasite undergoes genetic mutations or gene amplifications that enable it to survive and reproduce more effectively, resulting in reduced susceptibility to therapeutic agents.

Resistance to current antimalarial drugs can be attributed to various factors. These include the mutation rate of the parasite, the overall parasite load, the potency of the drug selected, treatment compliance, and inadequate adherence to malaria treatment guidelines (Paget-McNicol & Saul, 2001). Insufficient exposure of parasites to drugs can result from improper dosing, suboptimal pharmacokinetic properties, and counterfeit medicines (Müller et al., 2019). Poor-quality antimalarials, including falsified products without active pharmaceutical ingredients, may contribute to resistance by increasing the risks of hyperparasitemia, recrudescence, and hypergametocytopaenia. Additionally, using incorrect active pharmaceutical ingredients, such as substituting halofantrine for artemisinin, poses a risk of resistance and can go undetected without proper chemical analysis (Newton et al., 2011).

**Figure 1:**
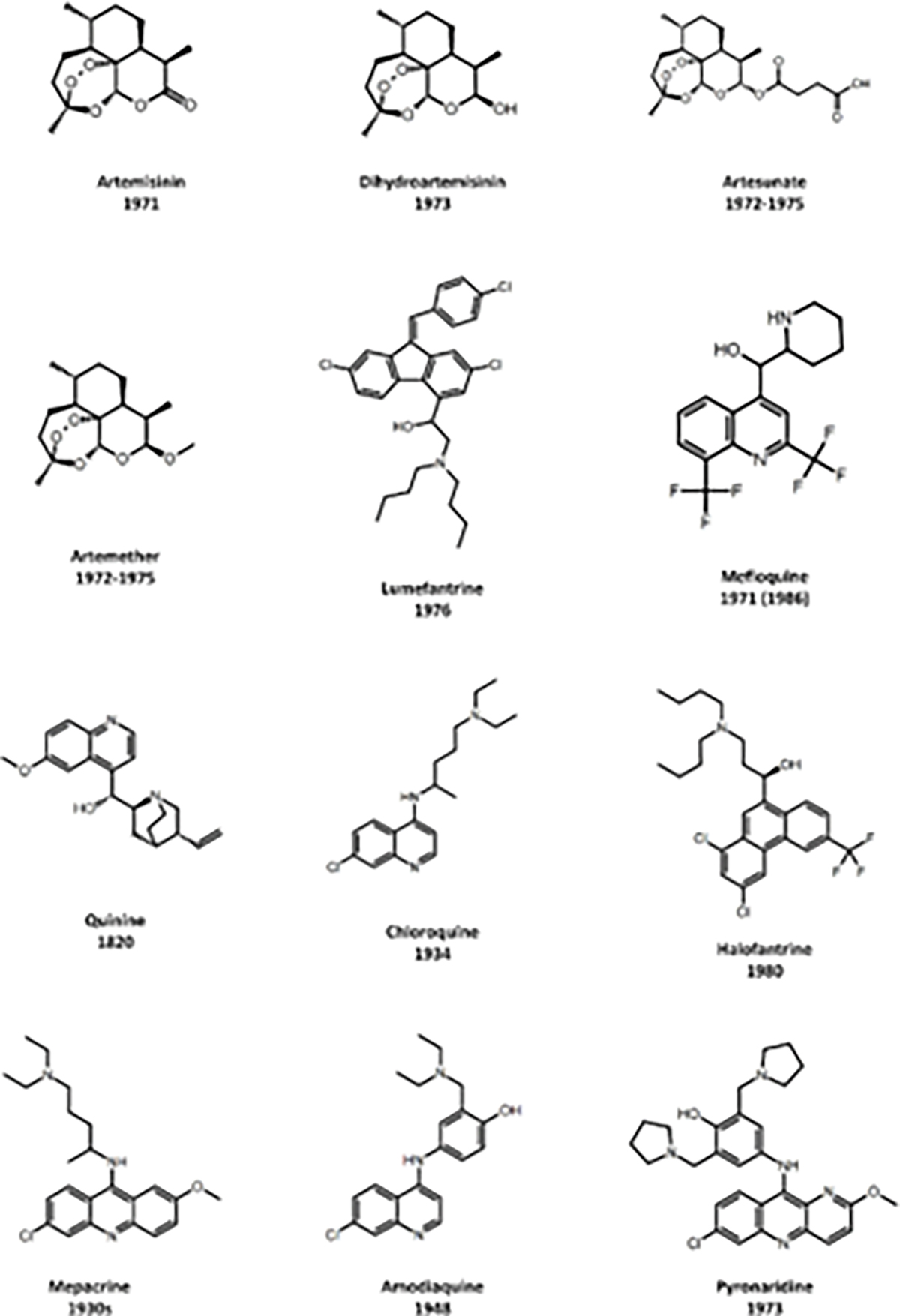
Chemical structures of antimalarial drugs that have previously or are currently in use and timeline of FDA approval.

Numerous drugs have previously received approval as antimalarials (refer to Figure 1 for a summary). However, the menacing *P. falciparum* parasites have developed resistance to most of these treatments. Artemisinin-Based Combination Therapy (ACT), which has been the recommended by WHO as frontline treatment for malaria since its introduction in the late 1990s and early 2000s, has demonstrated considerable efficacy. Nonetheless, there are growing concerns regarding the emergence of resistance to ACTs. Initial signs of partial resistance to ACT were observed in Greater Mekong Subregion (GMS) in Southeast Asia (Imwong et al., 2017; Jacob et al., 2021). Following this, documented instances of outright treatment failures involving artesunate-mefloquine (AS-MQ) and subsequently dihydroartemisinin-piperaquine (DHA-PPQ) surfaced. These failures have been attributed to confirmed resistance, both phenotypically and genotypically, impacting the artemisinin derivatives as well as their partner drugs (Phuc et al., 2017). While the widely used ACT-AL combination remains clinically effective in Africa, concerns are rising as partial resistance to ART has been confirmed in Rwanda. Additionally, recent findings from Uganda indicate a decline in *P. falciparum* susceptibility to lumefantrine and DHA, heightening worries about the potential evolution toward resistance and increased selective pressure on partner drugs (Siddiqui et al., 2021).

Recent advancements have significantly enhanced our understanding of the mechanisms by which *P. falciparum* parasites develop resistance to antimalarial drugs, particularly ART based therapies. Notably, mutations in the *kelch13* (K13) gene have been identified as a key factor contributing to partial resistance, enabling certain early ring-stage parasites to withstand ART treatment (Chhibber-Goel & Sharma, 2019; Stokes et al., 2021). Mutations in the *K13* gene lead to decreased protein levels and impair the internalization of hemoglobin, resulting in reduced amounts of Fe2+ heme that are essential for activating artemisinin-based therapies (ART). Consequently, patients undergoing ACT may experience delays in parasite clearance. Resistance to ACTs develops when both the ART derivative and the partner drug encounter resistance mechanisms (Masserey et al., 2022). In the Greater Mekong Subregion (GMS), resistance to dihydroartemisinin-piperaquine (DHA-PPQ) has emerged due to parasites that exhibit resistance to both components, a situation initiated by the amplification of *P. falciparum* genes that encode *plasmepsin 2* and *plasmepsin 3* (Imwong et al., 2017). Interestingly, *Pfplasmepsin* 2 gene amplification is primarily observed in parasites that carry the *PfKelch13*C580Y mutation, which is linked to piperaquine resistance, a partner drug in ACTs (Amato et al., 2017; Witkowski et al., 2017). The amplification *of Pfplasmepsin 2* appears to be a secondary adaptation to the drug pressure exerted by ACTs, following the selection of a dominant lineage characterized by the *PfKelch13*C580Y mutation (Imwong et al., 2017). This amplification enables the parasites to withstand piperaquine by enhancing the enzymatic degradation of hemoglobin within the parasite’s digestive vacuole, thus reducing the drug’s effectiveness. Notably, significant resistance to piperaquine is largely associated with mutant variants of the chloroquine (CQ) resistance transporter *P. falciparum* chloroquine resistance transporter (PfCRT). These mutations on PfCRT allow CQ to effluxed across the membrane into the cytosol, away from its heme/hemozoin target (Ross & Fidock, 2019). Studies propose the combination of drugs exerting opposing selective pressures on PfCRT and *P. falciparum* multidrug resistance protein 1 (PfMDR1) to suppress multidrug-resistant parasites (Siddiqui et al., 2021). These strategies, including adopting triple ACTs, aim to counter resistance and bolster effectiveness in regions reporting drug resistance. The spread of *P. falciparum* resistance to front line antimalarials highlights the need to develop new drugs with novel modes of action rapidly.

### Developing antimalarial therapies

Developing novel therapies to replace existing malaria treatments is demanding, necessitating specific criteria agreed upon by medical professionals, scientists, and patient advocates to assist WHO, Medicines for Malaria Venture (MMV) set Target Product Profiles (TPP), as well as Target Candidate Profiles (TCP) (Burrows et al., 2017; Burrows et al., 2014).

**Figure 2:**
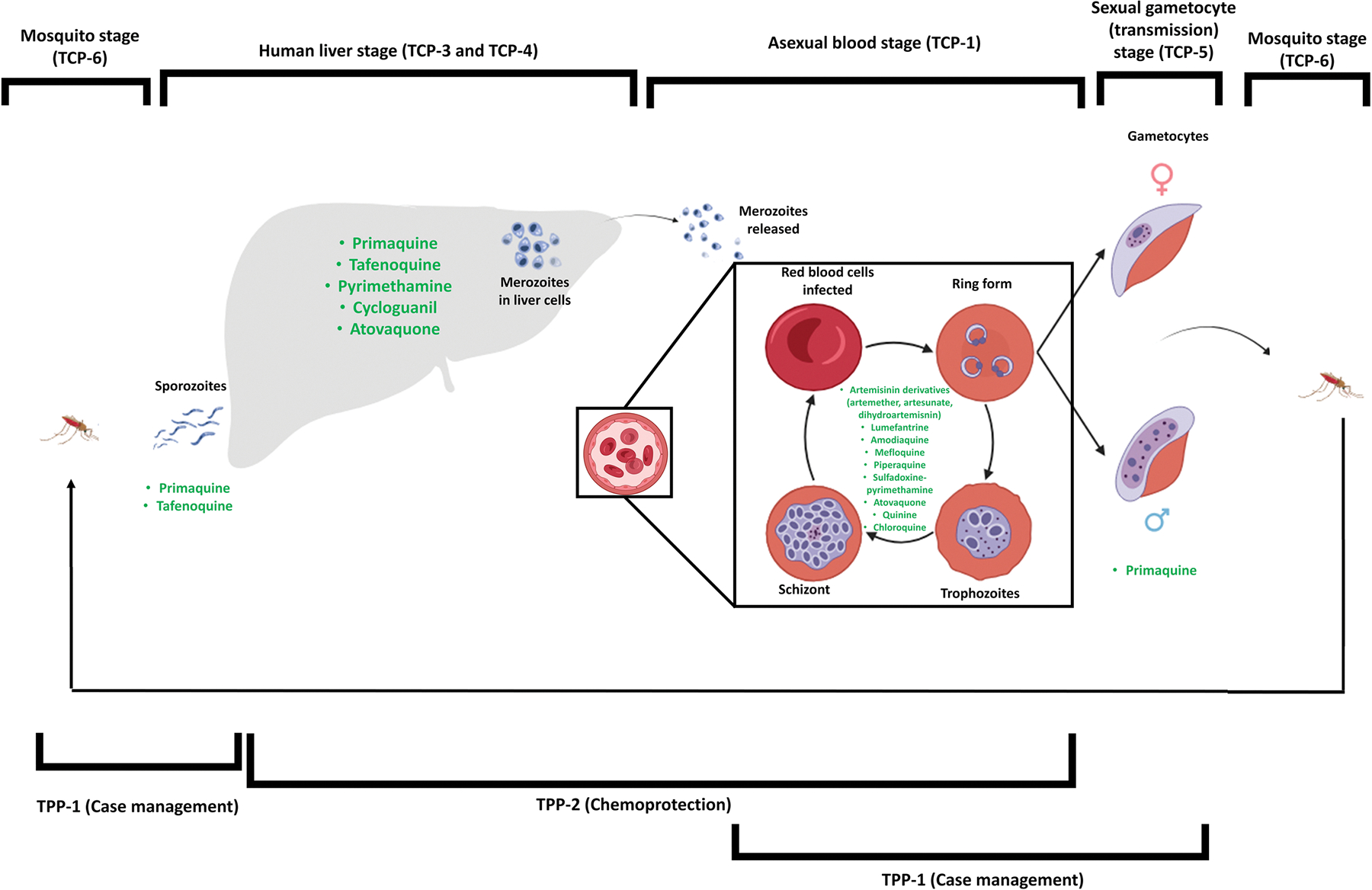
illustration of the different interventions of the TCPs and TPPs within the parasite life cycle adapted from (Siqueira-Neto et al., 2023). Current therapeutic interventions addressing TCP profiles are indicated with TCP-3 and TCP-4 curbing the liver stage, TCP-5 comprising the majority of antimalarials targeting the asexual blood stage of parasite development and TCP-6 transmission blocking agents inhibiting gametocyte activity.

TPP outline criteria for effective combination products that surpass standard care, comprising two categories: TPP-1 for treating uncomplicated malaria and TPP-2 for chemoprevention and prophylaxis (Siqueira-Neto et al., 2023). TCP defines essential characteristics for chemical molecules used in malaria therapy (Burrows et al., 2017). These TCPs are listed in Table 1 and current malaria intervention indications in Figure 2. Furthermore, TPPs addressing new therapeutics for severe malaria have been updated, wherein the near-term goal involves a combination of approved antimalarials to current parenteral and rectal artesunate-based therapies, allowing for mitigation against artemisinin partial resistance while reducing the risk for further selection and dissemination of resistant parasites (Achan et al., 2024). The long term would involve developing artemisinin alternatives for identifying new combinations (Achan et al., 2024).

The complexity of malaria necessitates considerations of several factors which include drug resistance, safety, and pharmacokinetic-pharmacodynamic relationships in designing new medicines. Key objectives include developing potent antimalarial compounds with diverse mechanisms of action and extended half-lives for monthly dosing. Long-acting injectables are also considered, provided wide distribution and lasting protection can be achieved (Achan et al., 2024). Chemical stability in tropical conditions and pediatric tolerability are crucial, demanding robust pharmacovigilance and population-specific genetic analysis (Siqueira-Neto et al., 2023). Formulations suitable for children and reproductive safety, particularly in early pregnancy, are imperative due to the high risk faced by pregnant women.

Furthermore, the discovery process for antimalarials involves identifying single candidate drugs before evaluating optimal combinations to prevent resistance and ensure compliance. The challenge is compounded by the prevalence of disease among impoverished populations in sub-Saharan Africa, prompting the need for free access through public programs and partnerships like the MMV. MMV collaborates with various experts and stakeholders to develop and deliver new antimalarial products, focusing on enhancing current medications to guide future treatment goals. While prioritizing safety and enhancing ease of dosing, research endeavours in drug discovery has developed numerous novel compounds with new modes of action. These are in late stages of clinical development, representing the most advanced progress in this field (Table 2).

There is a pressing need to feed the drug discovery pipeline with new chemotypes to address the problem of malaria. Most compounds that have been developed and progressed to treat malaria have mainly been identified by phenotypic screening. There is a need to explore target-based drug discovery approaches to potentially address the likelihood of drug resistance emergence *in vitro*.

### The role *P. falciparum* Hsp90 in the life cycle of the parasite

**Figure 3:**
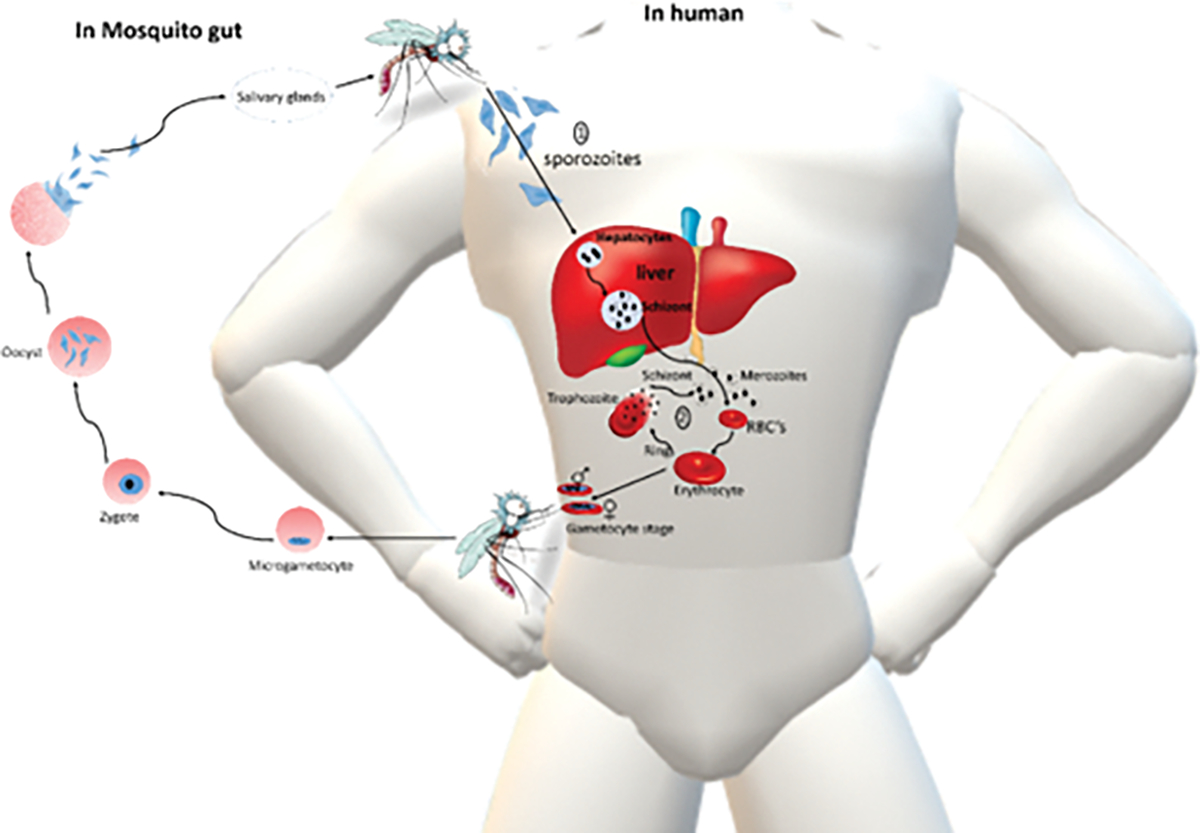
Life cycle of *P falciparum*. 1, migration of sporozoites through the blood stream after a bite from the infected female *Anopheles* mosquito to invade hepatocytes. 2, the sporozoites differentiate and mitotically divide into thousands of merozoites which are subsequently released and enter the intra-erythrocytic cycle (asexual blood stage), while a sub-population of the merozoites switch to sexual development gametocytes. 3, the gametocytes are ingested by female Anopheles mosquito and undergo development, which results in sporozoites in the salivary glands of the mosquito. Figure adapted from Tankeshwar, 2021.

The life cycle of *P. falciparum* occurs between the human host and mosquito vector (Summarized in Figure 3). Malaria transmission begins when an infected *Anopheles* mosquito bites a host, injecting sporozoites into the bloodstream. These sporozoites invade liver cells and undergo a reproductive phase, forming schizonts containing merozoites over 1–2 weeks. Mature schizonts burst, releasing merozoites into the bloodstream. In the case of *P. falciparum*, this hepatic phase lasts about five days. Merozoites invade red blood cells, initiating a cycle of asexual reproduction. Within 20–24 hours, trophozoites and schizonts develop from merozoites inside red blood cells, leading to their rupture and release of more merozoites (Bannister & Mitchell, 2003). This cycle repeats, causing erythrocytic schizogony. Ruptured schizonts release parasites and red blood cell contents into the bloodstream, causing malaria symptoms to manifest (Aly et al., 2009; Bannister & Mitchell, 2003). To ensure successful infection, *P. falciparum* relies on heat shock proteins to maintain its cellular integrity and to resist environmental stress.

The *P. falciparum* parasite faces significant environmental insults during its life cycle when transitioning from a cold-blooded insect vector to a warm-blooded mammalian host (Shahinas & Pillai, 2021). Malaria-infected individuals experience periodic fever every 48 hours, which can increase their body temperature to an astounding 42°C (Shahinas & Pillai, 2021). This sudden rise in temperature is a significant stressor for the parasite’s cellular machinery, which must adapt quickly to survive. Roughly 2% of *P. falciparum*’s genes produce molecular chaperones (Acharya et al., 2007). Studies have shown that when *P. falciparum* is grown *in vitro* at temperatures of 39°C and 41°C, the transcription of *P. falciparum* (PfHsp90) is elevated three to fourfold (Kumar et al., 2003).

Distinct transcriptome levels of Hsp90 genes have been correlated with clinical manifestations of malaria (Neckers & Tatu, 2008). PfHsp90 is expressed at all blood stage phases of the parasite life cycle: ring, trophozoite, and schizont. Inhibition of PfHsp90 function by pharmacological agents has been shown to abrogate parasite growth in the blood and in the liver, and early gametocyte life stages (Mansfield et al., 2024; Murillo-Solano et al., 2017; Posfai et al., 2018; Wang et al., 2016). PfHsp90 and PfCRT are located on the same gene clusters, possibly regulated by the same transcription mechanism. Additionally, PfHsp90 has been implicated in drug resistance by showing that it interacts directly with the PfCRT (Shahinas et al., 2013). Therefore, PfHsp90 is an attractive drug target for developing multi-stage targeting, effective and dependable antimalarial drugs that inhibit *P. falciparum* infection and growth while reducing the possibility of developing resistance to treatment. This review focuses on the potential of PfHsp90 as an attractive drug target for malaria.

### *P. falciparum* Hsp90 Isoforms

**Figure 4:**
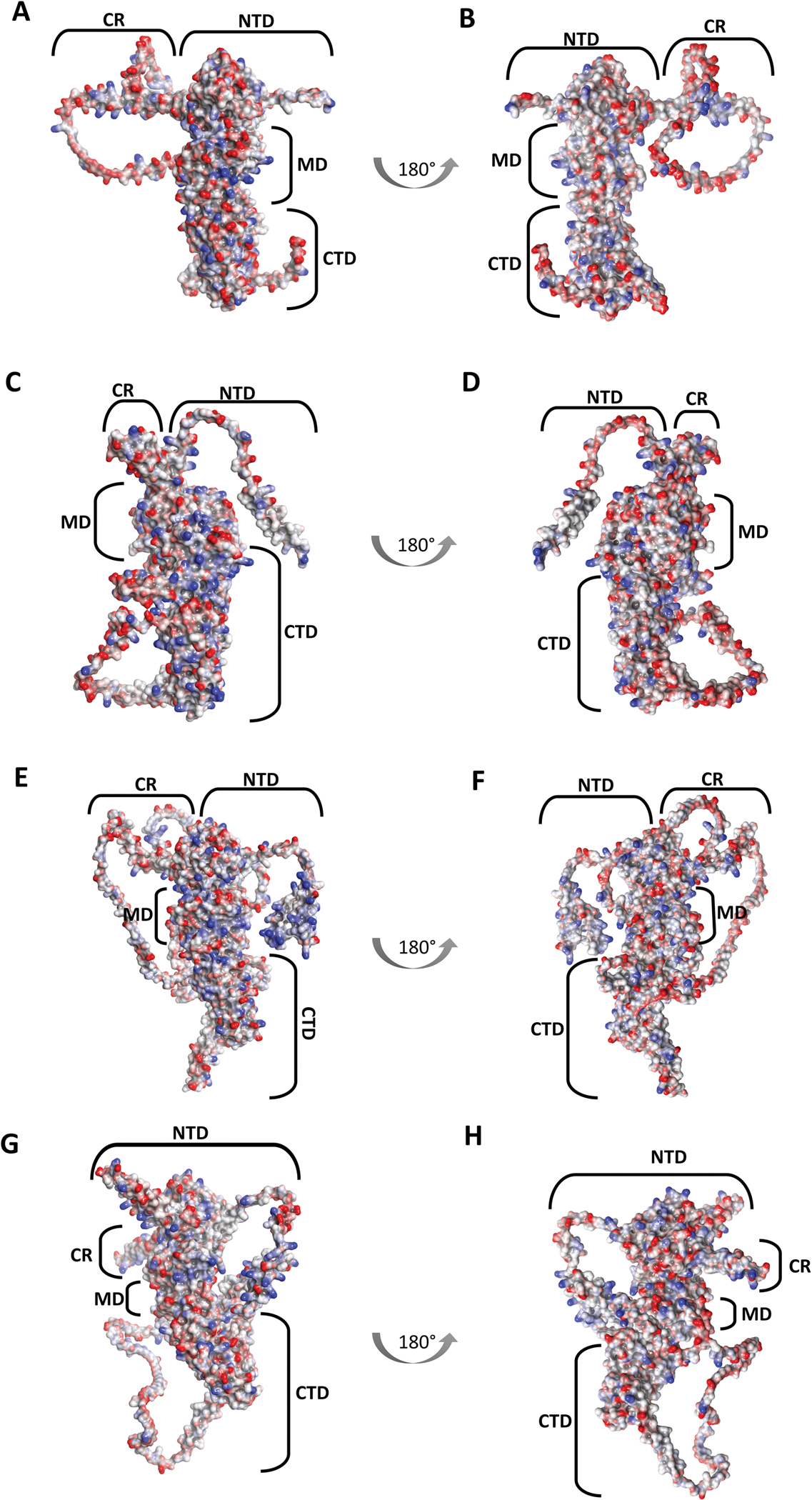
Three-dimensional structures of the PfHsp90 isoforms obtained from Alphafold (https://alphafold.ebi.ac.uk/), highlighting the electrostatic potential of each isoform. The diagram indicates the front and back viewpoints of each isoform: PfHsp90 (A and B), PfGRP94 (C and D), PfTRAP-1 (E and F) and PfHsp90_A (G and H), with the domains of each isoform indicated as the N-terminal domain (NTD), Middle domain (MD) and C-terminal domain (CTD). The charged linker region is annotated as CR.

As a vital area of research in the pursuit of novel treatments for malaria, particularly those that could counteract antimalarial drug resistance, the Hsp90 isoforms encoded by *P. falciparum* have been the focus of several important studies (Banumathy et al., 2003; Everson et al., 2021; Mansfield et al., 2024; Murillo-Solano et al., 2017; Posfai et al., 2018; Shahinas et al., 2013; Shahinas & Pillai, 2021; Wang et al., 2014, 2016). As shown in Figure 4, the genome of *P. falciparum* encodes four Hsp90 isoforms, each residing in different cellular compartments: PfHsp90 (PlasmoDB accession number: PF3D7_0708400), PfGRP94 (PF3D7_1222300), PfTRAP-1 (PF3D7_1118200), and PfHsp90_A (PF3D7_1443900), which are localized in the cytoplasm, endoplasmic reticulum, mitochondria, and apicoplast, respectively (Silva et al., 2020). The cytosolic isoform, PfHsp90, shares a higher conservation with the human counterpart, human Hsp90 (HsHsp90), sharing a 75% sequence identity in the N-terminal domains (NTDs) (Acharya et al., 2007). In comparison, the NTDs of the *P. falciparum* isoforms display lower respective conservations. The NTD of PfHsp90 shares some conservation with the NTDs of PfGRP94 (42%) and a weak conservation with apicoplast PfHsp90 (PfHsp90_A) (30%) and PfTRAP-1 (25%) which possess inserts at the NTD. In addition, there are numerous possibilities for the selective targeting of each isoform, as evidenced by the comparisons of the full-length structures of each isoform (Figure 4 rendered based on electrostatic potential), which indicate that the chaperone differences are primarily located on the charged linker region and the CTD.

## Comparisons of the N-terminal domains of *P. falciparum* Hsp90 isoforms

**Figure 5:**
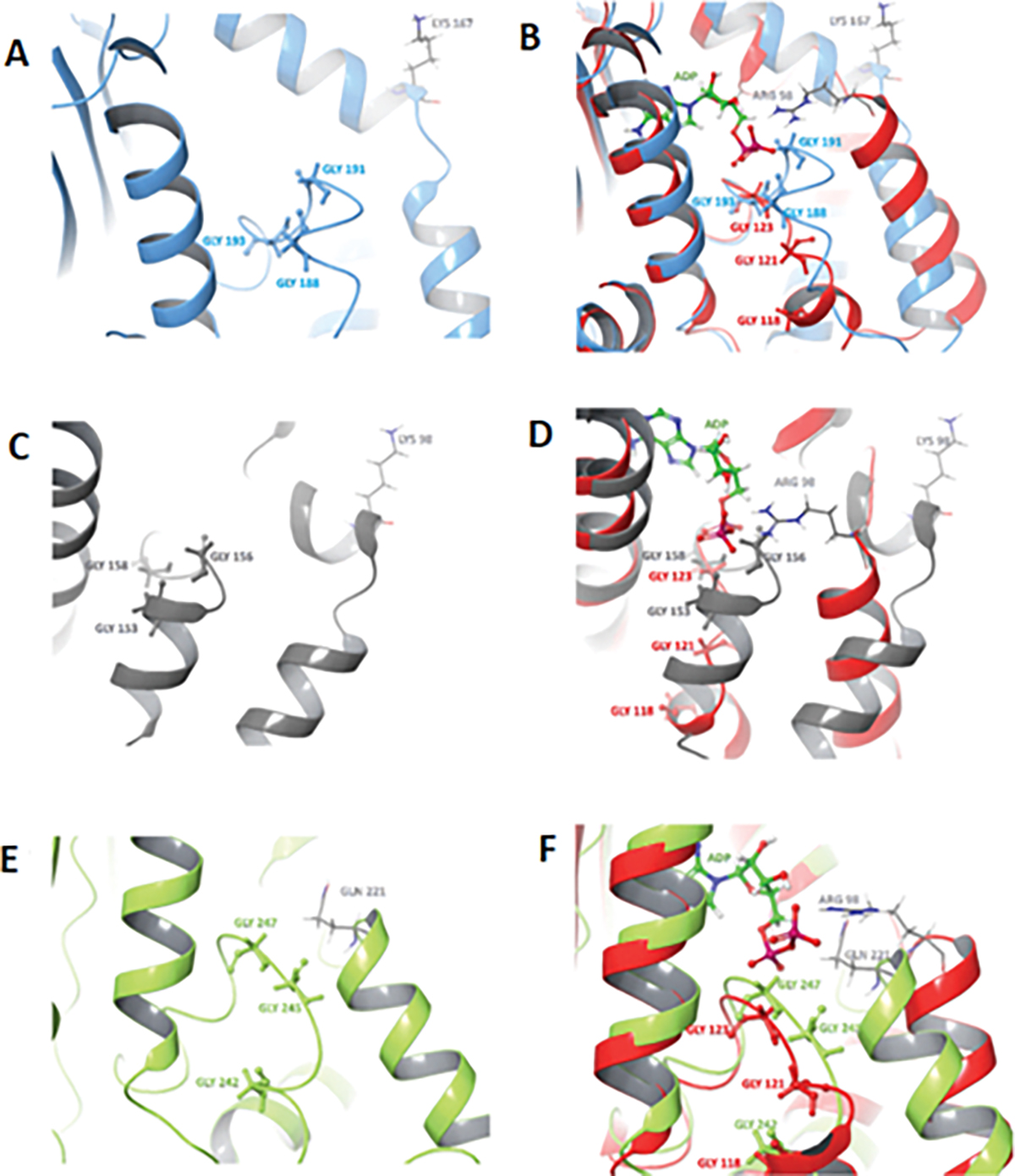
Binding pockets and glycine-rich loops (GHL) of the NTDs of (A) PfGRP94 (light blue, PDB, ID: 3PEH) consisting of Gly188, Gly191 and Gly193 (light blue), (B) PfTRAP1 (grey, modelled using the template of *Homo sapiens* TRAP1, PDB, ID: 7C04) consisting of Gly153, Gly156 and Gly158 (yellow), and (D) PfHsp90_A (light green, PDB, ID: 3IED) consisting of Gly242, Gly245 and Gly247 (light green). Protein structure alignments of the binding pocket of PfHsp90 (red) superimposed onto those of (B) PfGRP94 (light blue), (D) PfTRAP1 (grey) and (F) PfHsp90_A (light green).

We compared the primary amino acid sequences of N-terminal (ATP/drug binding site) PfHsp90 with the organelle Hsp90 isoforms in *P. falciparum* and HsHsp90. Both PfTRAP-1 and PfHsp90_A possess >30 amino acids insert on the NTD which are rich in charged (Lys/Glu) and Asn residues, respectively. The roles of these unique insertions on the isoforms of *P. falciparum* need to be investigated further. When looking at the ATP binding residues, only one substitution was found in the isoforms compared to cytosolic Hsp90. Specifically, the Arg98 of PfHsp90 is replaced by Lys167, Kys98, and Gln221 in PfGRP94, PfTRAP-1, and PfHsp90_A, respectively. This difference is important for testing the hypothesis that Arg98 is necessary for selectivity (Shahinas et al., 2012). Our preliminary structural comparison indicates that the glycine-rich region of PfGRP94 closely resembles that of human Hsp90 (see Figure 5A, B), whereas PfTRAP-1 appears to be the most distinct, with an alpha-helix instead of a loop in the glycine-rich region (see Figure 5C, D). Despite the differences at the sequence level, the glycine-rich region of PfHsp90_A seems to most closely resemble the straight conformation of PfHsp90 (see Figure 5E, F) when the insert is removed. We believe that these differences are exploitable for designing species selective inhibitors by rational design strategies.

### PfHsp90 as a drug target

The laudable efforts of Hsp90 targeting in eukaryotic organisms have inspired repurposing of the compounds for parasitic disease. However, several lessons have been learned from currently available Hsp90 inhibitors, especially from cancer research. For instance, GDA, although highly effective and with selective affinity towards cancerous cells with minimal effect on normal cells, has been reported to have hepatotoxic side effects (Neckers et al., 1999). Known inhibitors of human Hsp90 have recently been reviewed (Rastogi et al., 2024), most of which have had limited its pharmaceutical applications due to toxicity, pan-inhibition or insufficient funding. It noteworthy indicated that pimitespib, while still limited to Asia, has shown some promising results with low toxicity.

Among the four isoforms of Hsp90 in *Plasmodium falciparum*, PfHsp90 has garnered the most attention as a potential drug target. Anti-cancer inhibitors of Hsp90 have been shown to inhibit parasite growth *in vitro* and disrupt the biochemical function of PfHsp90 (Pallavi et al., 2010). This supports the validity of PfHsp90 as a potential drug target, which was evidenced by pre-clinical studies using mice infected with *P. berghei*. In these studies, inhibitors such as 17-AAG and PUH-71 were found to cure mice models (Shahinas et al., 2013). However, while existing Hsp90 inhibitors demonstrate antimalarial effects, their limitations in clinical trials necessitate the discovery of new molecules that are selective for PfHsp90 for successful drug development.

The significance of PfHsp90 has been emphasized in numerous studies (Banumathy et al., 2003; Corbett & Berger, 2010; Mafethe et al., 2023; Shahinas et al., 2013; Shahinas & Pillai, 2021; Wang et al., 2014, 2016),. PfHsp90 exhibits a high sequence identity with HsHsp90, prompting an investigation into their biochemical and structural differences with the goal of exploiting these variations to design PfHsp90-selective inhibitors. The crystal structures of the N-terminal domain (NTD) of PfHsp90 (PDB code: 3K60) and HsHsp90 (PDB code: 1YBQ) in complex with ADP has been resolved, providing a foundation for comparative analysis analysis (Corbett & Berger, 2010). Notably, several key residue differences, including Ala38, Met84, and Ile173 in PfHsp90, contribute to a constricted architecture of the binding pocket. Furthermore, an Ala38Ser substitution leads to increased hydrophobicity within the binding pocket of PfHsp90 (Corbett & Berger, 2010).

**Figure 6:**
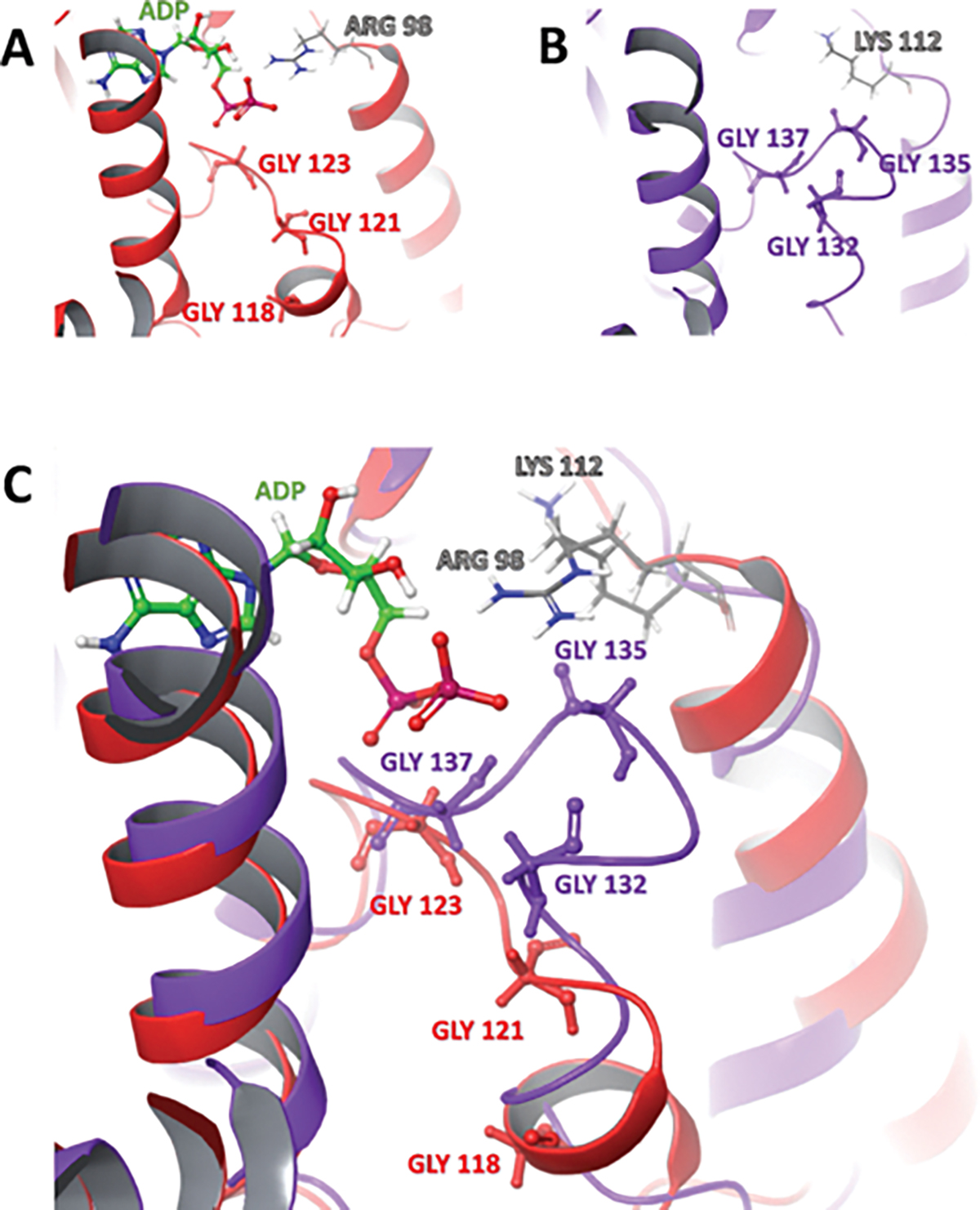
Binding pockets and glycine-rich loops (GHL) of the NTDs of (A) PfHsp90 (red, PDB, ID: 3K60) consisting of Gly118, Gly121, and Gly123 (red) and (B) HsHsp90 (purple, PDB, ID: 1BYQ) consisting of Gly132, Gly135 and Gly137 (purple). (C) Protein structure alignments of the binding pocket of PfHsp90 (red) superimposed onto that of HsHsp90 (purple).

The binding pockets of HsHsp90α and β, as well as PfHsp90, consist of a glycine-rich loop (GHL), which is more compacted in HsHsp90 due to the clipping together of residues Gly135 and Gly114 (Figure 6A) and exists in a straight conformation in PfHsp90 due to the disconnecting Gly121 from Gly100 (Figure 6B). This structural variation opens a hydrophobic cavity in PfHsp90’s binding pocket, potentially allowing the accommodation of small molecules (Wang et al., 2014).

The fundamental differences in the binding pocket properties are crucial for developing selective inhibitors targeting PfHsp90. However, these efforts would prove to be prudent when using rational design strategies, as shown by Wang and colleagues (2014) through the identification of three key substitutions: Ala38Ser, Arg98Lys, and Ile173Val. Although the Ala38Ser and Ile173Val substitutions may not provide any significant changes to the binding pocket architecture (Wang et al., 2014), the Arg98Lys may be more favorable as the guanidinium of arginine, with its planar conformation, promotes the formation of multiple hydrogen bonds that bind aromatic moieties of candidate drugs.

PfHsp90 and HsHsp90 display distinct biochemical activities, with a notable difference being that PfHsp90 has an ATP affinity that is six times higher than that of HsHsp90 (Pallavi et al., 2010). Additionally, these two chaperones respond differently to geldanamycin (GDA), as PfHsp90 demonstrates a stronger affinity for this NTD inhibitor compared to HsHsp90 (Table 3). Research by Banumathy and colleagues (2003) has shown that GDA effectively halts the progression of parasites from the ring stage to the trophozoite stage. Moreover, Kumar and colleagues (2003) reported a synergistic effect when GDA was administered alongside chloroquine (CQ).

### Small molecule selective inhibitors of the N-terminal of PfHsp90

The first selective inhibitor of PfHsp90 to be described was Harmine which was identified from high-throughput screening as an ATP-competitive inhibitor (Shahinas et al., 2010). Harmine specifically inhibits PfHsp90 without affecting HsHsp90, demonstrating IC_50_ values in the nanomolar range for drug-sensitive (3D7) and chloroquine-sensitive (W2) parasite strains (Shahinas et al., 2012). Furthermore, harmine and chloroquine were found to produce synergistic effects (Shahinas et al., 2012). These findings were supported by a later study conducted by Posfai and colleagues (2018), confirming the selective binding of harmine to PfHsp90 and no binding towards HsHsp90. One limitation of harmine is its low binding affinity (see Table 3, K_D_ = 40.0 μM) for PfHsp90. Optimization efforts led to compounds 17A and 21A; although their binding affinities remain undetermined, they are believed to bind PfHsp90 with improved affinities (Bayih et al., 2016). Additionally, both 17A and 21A inhibited the growth of the W2 strain of the parasite without exhibiting cytotoxicity towards HepG2 and HeLa cell lines (Bayih et al., 2016).

Rational design strategies have also been applied to develop selective inhibitors of PfHsp90. Everson and colleagues (2021) screened drug-like compounds from the ZINC15 database to identify PfHsp90 inhibitors with binding energies of ≥11.0 kcal/mol. After applying selection criteria, 12 compounds were identified and tested on *P. falciparum* 3D7 parasites. Among these, three compounds—CP-6, CP-7, and CP-10—exhibited single-digit micromolar activity or better (Everson et al., 2021). However, due to the absence of biochemical assays, potential off-target effects could not be ruled out. These off-target effects could involve unintended interactions with other parasitic proteins, leading to unknown toxicities or diminished efficacy. It remains unclear whether the anti-*Plasmodium* activities of these compounds are solely due to their primary interactions with PfHsp90 or if they also affect other parasitic proteins. Nevertheless, the results indicate a possibility for obtaining selective inhibitors of PfHsp90.

Using ligand-based drug design methods, we explored compounds from the Enamine and MMV malaria box databases for potential binding to PfHsp90 (Mafethe et al., 2023). Our study employed a pharmacophore model, created from selective PfHsp90 inhibitors found in literature (Wang et al., 2016), to search these databases for chemically diverse candidates. A virtual screening protocol, utilizing extra-precision docking, assessed the ability of these inhibitors to bind to the PfHsp90 binding pocket, resulting in the identification of compounds FM2, FM4, and FM6 (Table 3), which directly interact with the selectivity-conferring residue Arg98 (Mafethe et al., 2023). These compounds exhibited promising activity against *P. falciparum* (see Table 3), although FM4 and FM6 require further optimization due to their noted toxicities towards HepG2 cells (Table 3). Compound FM6 displayed a strong binding affinity (in the nanomolar range) towards PfHsp90 but was toxic to HepG2 cells. Conversely, compound FM2 did not exhibit cytotoxicity; however, it showed weak affinity for PfHsp90 (Mafethe et al., 2023), potentially due to non-specific binding or interactions with PfHsp90 isoforms in the endoplasmic reticulum and mitochondria. This highlights the challenges in designing effective PfHsp90 inhibitors.

The lack of correlation between the binding affinities of PfHsp90 inhibitors and their potency against *P. falciparum* raises concerns about possible off-target interactions with other parasitic proteins. Hsp90s are part of the GHKL superfamily of proteins, which also includes DNA topoisomerase II, DNA mismatch repair enzymes (MutL), and histidine kinases (Dutta & Inouye, 2000). A key characteristic of this superfamily is the unconventional Bergerat ATP-binding fold. Our understanding of the structural similarities within the GHKL family of proteins is still evolving in eukaryotic organisms and is particularly underexplored in *P. falciparum*. Insights gained from anti-cancer drug discovery aimed at Hsp90 suggest that selective inhibitors are often screened against topoisomerase II and a panel of 22 kinases to confirm direct interaction with the target (Dernovšek et al., 2024). The observed disconnect between binding affinity and potency against *P. falciparum* indicates the need for further investigations, particularly in assessing the potential pan-inhibition of other Hsp90 isoforms and/or GHKL family members in *P. falciparum*.

### Natural products and ATP-mimmetics inhibiting the N-terminal domain of PfHsp90

Researchers have been investigating natural products that could serve as inhibitors of PfHsp90, which presents a promising avenue of study. A study conducted by Daniyan and Ojo (2019) utilized molecular docking techniques to propose that several phytochemicals derived from *Azadirachta indica* have the potential to act as PfHsp90 inhibitors, underscoring the necessity for experimental validation of these predictions. Both PfHsp90 and PfHsp70–1 (PlasmoDB accession number: PF3D7_0818900) demonstrate the ability to bind ATP, suggesting that inhibitors with structures resembling ATP, such as ATP mimetics or purine analogues, could effectively target both PfHsp90 and PfHsp70–1 (Cockburn et al., 2011). In another study, Nndwammbi et al. (2024) explored the repurposing of known PfHsp70–1 inhibitors, namely ursolic acid acetate (UAA) and iso-mukaadial acetate (IMA), for use as PfHsp90 inhibitors. UAA showed substantial binding affinity to PfHsp90 in the micromolar range (Table 3), while IMA exhibited a lower binding affinity (Nndwammbi et al., 2024).

Although our research did not concentrate on natural products, we recently employed active learning models to identify ATP mimetics (purine and pyrimidine-based nucleosides) as potential inhibitors of PfHsp90 (Matlhodi et al., 2024). The analogues, which exhibited moderate anti-*Plasmodium* activity and appreciable selectivity indices against mammalian cells, hold considerable promise for the future of PfHsp90 research. While the activities of these ATP mimetics against Hsp90 isoforms residing in various parasite compartments and GHLK have yet to be examined, we suspect the existence of some off-target effects. Previous studies have indicated that optimizing ATP mimetics may yield selective inhibitors with exceptional potency against pathogenic organisms (Skácel et al., 2023). As such, identifying these ATP mimetics as potential inhibitors of PfHsp90 provides an encouraging starting point for future optimization efforts.

### Targeting PfGRP94 and PfHsp90_A in *P. falciparum*

The domain structures of PfGRP94 and PfHsp90 are similar. However, in contrast to PfHsp90, PfGRP94 is reported to have a weaker/non-specific binding affinity to GDA in the millimolar range (Murillo-Solano et al., 2017). Although a comparative analysis of the structures of the binding pockets of PfHsp90 and PfGRP94 has not been conducted, a multiple sequence alignment with PfHsp90, PfGRP94, and HsHsp90α and β revealed a unique substitution of Arg98 in PfHsp90 that is not observed in PfGRP94 (Stofberg et al., 2021). This divergence may offer a pathway to explore for developing isoform-specific inhibitors.

Murillo-Solano and colleagues (2017) assessed the potential of the GDA derivatives, 17-AAG and 17-DMAG, to act as inhibitors against PfGRP94. The study demonstrated that 17-AAG had a higher binding affinity towards HsHsp90 than PfGRP94, while 17-DMAG displayed a higher binding affinity towards PfGRP94 (Murillo-Solano et al., 2017). The respective binding affinities of these compounds against PfGRP94 and HsHsp90 are tabulated (Table 4) below.

The apicoplast-localized isoform of Hsp90 in *P. falciparum* has received little attention regarding its biochemical function. Regardless, functional similarities can be inferred due to its similarity to PfGRP94 as proteostasis proteins that reside in oxidizing organelles (Stofberg et al., 2021). The apicoplast is a membrane-bound organelle located within intracellular parasites of the Apicomplexa phylum, including *Plasmodium* and *Taxoplasma* (Chakraborty, 2016). The survival of *P. falciparum* parasites has been linked to apicoplast function. The disruption of the apicoplast’s segragation mechanism resulted in viable parasitic cells incapable of progressing into division (He *et al.,* 2001; Dahl & Rosenthal, 2007).

In the apicoplast, PfHsp90_A is expressed under normal physiological conditions and in response to stress (Mallo *et al.,* 2018; Sheiner *et al.,* 2013). With an NTD, a CR, a MD and a CTD, PfHsp90_A shares similarities with other isoforms regarding domain structures. Incorporated into the NTD of PfHsp90_A is a signal peptide that regulates its translocation across the apicoplast membranes and also possesses an insert mainly charged (Glu and Lys) of about 28 amino acids. Furthermore, the CTD includes a KTLL sequence, similar to that of GRP94, which may function as a retention signal sequence (Henry *et al.,* 2014). Notably, derivatives of RA, through hydrogen bonds with carboxylate groups of Asn107, Asp149, Lys168 and Thr245, displayed selectivity towards GRP94 (Crowley *et al.,* 2016; Mishra *et al.,* 2016). Interestingly, these residues are conserved in PfHsp90_A, wherein a tempting speculation can be made that PfHsp90_A may be susceptible to these RA derivatives.

### The mitochondrial PfTRAP-1

Compared to all isoforms of Hsp90 in *P. falciparum*, PfTRAP-1 is the least studied and least conserved. We found that it harbors significant sequence and structural differences (Figure 5 E,F). The inhibitor, Radicicol (RA), was found to halt parasite development in the schizont stage, possibly by inhibiting mitochondrial replication (IC_50_ = 9.029 μM), was repurposed for exposure to *P. falciparum* (Sureshkumar et al., 2014). Additionally, RA was postulated to exhibit antimalarial effects through its interaction with PfTRAP-1 or topoisomerase VIA/VIB, which both contain a Bergerat fold motif that binds to ATP (Chalapareddy et al., 2016)

### Protein-protein and CTD Inhibitors

Most research on Hsp90 has primarily centered on ATP-competitive inhibitors of the NTD. However, in cancer research, these inhibitors have exhibited certain limitations, such as the induction of pro-survival heat shock responses and insufficient selectivity across isoforms. Given the significance of Hsp90 as a drug target, alternative inhibition strategies have focused on the C-terminal domain (CTD) to disrupt protein-protein interactions within the Hsp90 complex. Unlike the mammalian system, both CTD and protein-protein interactions inhibitors have received less attention in *P. falciparum*.

## CTD inhibitors

**Figure 7:**
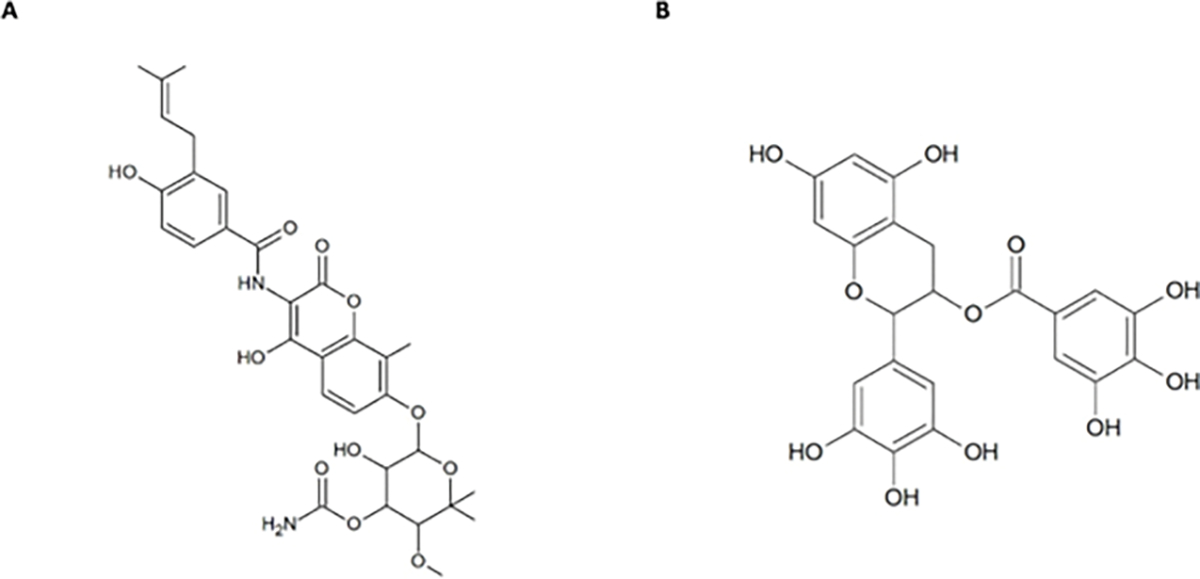
Chemical structures of Novobiocin (A) and EGCG (B).

The focus of Hsp90 studies has primarily been directed towards N-terminal domain inhibitors. A point of contention with the use in NTD inhibitors is the initiation of the heat shock response, and the consequent overexpression of heat shock proteins, which has led to the failure of many Hsp90 inhibitors in clinical trials (Amatya & Blagg, 2023). Therefore, CTD inhibitors have been postulated to circumvent this response. Within the CTD of Hsp90, a second ATP-binding site was discovered that could bind ATP, novobiocin and epigallocatechin-3-gallate (EGCG) shown in Figure 7 (Yin et al., 2009). Unlike NTD inhibitors that halt Hsp90 activity by disrupting ATPase activity, CTD inhibitors disrupt the interaction with tetratricopeptide repeat (TRP)-containing co-chaperones (Li & Luo, 2023; Scheufler et al., 2000). As such, CTD inhibitors may offer another pathway towards PfHsp90 inhibition.

Novobiocin was the first inhibitor to be discovered, binding to the second ATP-binding site near the dimerization site of Hsp90 (Garnier et al., 2002; Marcu et al., 2000). Novobiocin competes with ATP at the binding site on the CTD, disrupting the binding of co-chaperones that bind to this region (Marcu et al., 2000). However, as a consequence of Novobiocin’s high affinity towards Hsp90 (IC_50_ = 700 μM), analogues with higher potency were developed, including 4-deshydroxynovobiocin (DHN1) and 3′-descarbamoyl-4-deshydroxynovobiocin (DHN2), displaying IC_50_ values of 7.5 μM and 0.5 μM, respectively (Burlison et al., 2006).

Another CTD inhibitor, EGCG, an active ingredient in green tea, has been proposed to inhibit Hsp70 by binding to the ATP binding site (Brierley-Hobson, 2008), and has been demonstrated to exhibit antiplasmodial activity (Zininga et al., 2017). Yin and colleagues (2009) assert that EGCG binds to Hsp90 at or near CTD. It is tempting to speculate that EGCG may interact with PfHsp70–1 and PfHsp90, leading to the observed antiplasmodial activity. However, this idea requires experimental validation. Additionally, evidence suggests that CTD inhibitors bind to and inhibit Hsp90 without activating the pro-survival heat shock response. (Armstrong et al., 2016; Eskew et al., 2011; Liu et al., 2015).

### Co-chaperones of Hsp90 as drug targets

**Figure 8:**
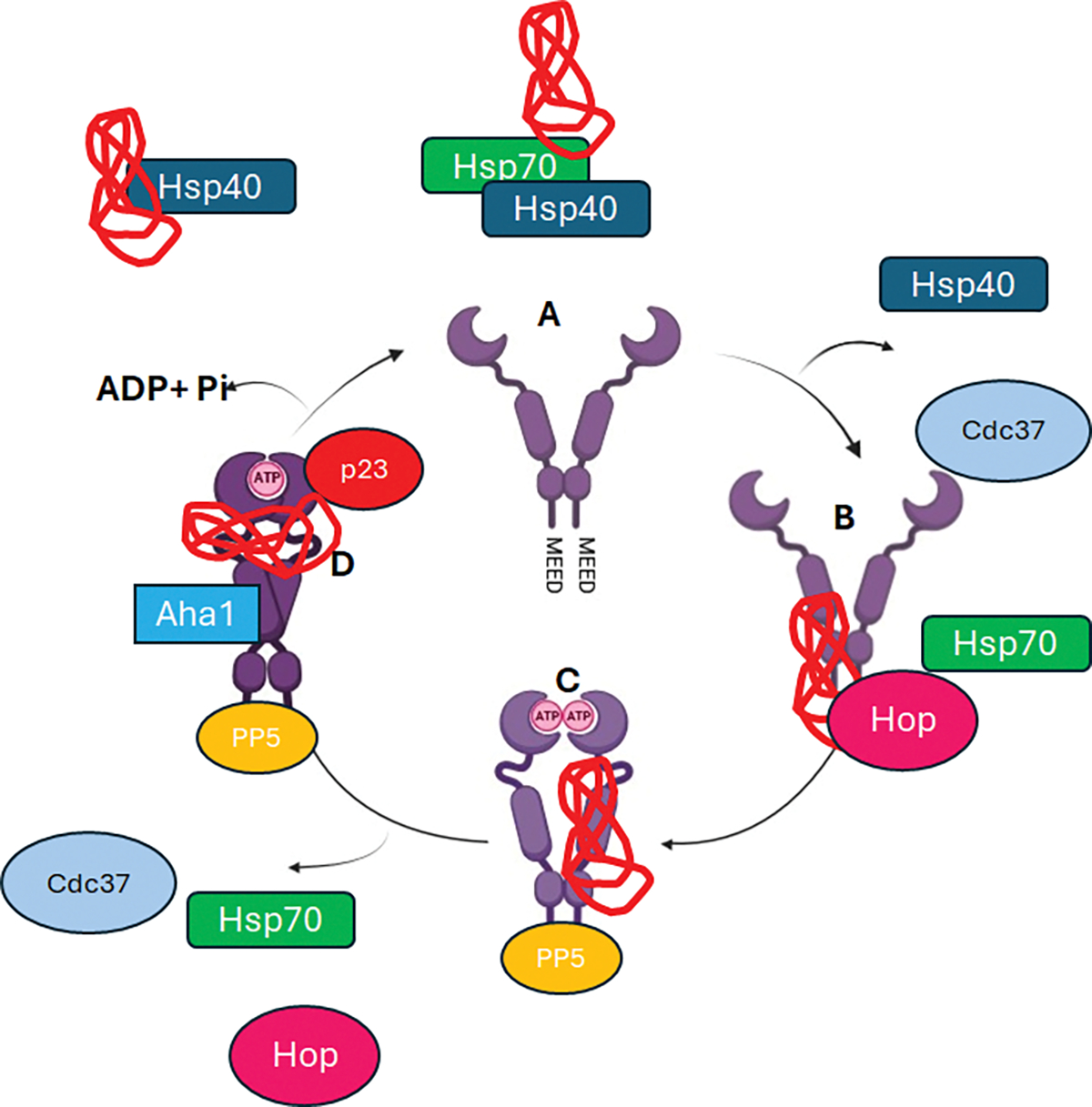
The illustration above depicts the Hsp90 chaperone cycle, a cycle necessary for the folding of client proteins. Hsp70/40 complex binds to the nascent client protein and these serve as a ‘transport” for the protein to the open conformation Hsp90 in A. B-The client protein bound to the Hsp70 is then associated with the Hsp90 via the Hop Cdc37 and immunophilin like proteins bind to intermediate Hsp90 complex. C-dissociation of the Hsp70 and Hop proteins and nucleotide (ATP) binding onto the Hsp90 NTD, conformational switch soon follows from open state to closed state. D-Hsp90 is bound by p23 and Aha1 proteins ATP hydrolysis ensues, results in energy release to fold the nascent unfolded protein, ADP and Pi are by-products. Immunophilins, cdc37, p23 and folded protein dissociate from the Hsp90. (Illustration adapted from Edkins & Boshoff, 2021).

Hsp90 plays a crucial role in the folding, activation, and stabilization of over 300 client proteins that are involved in various biological processes, including signal transduction, cellular trafficking, chromatin remodeling, cell growth, differentiation, and reproduction. To fulfill this role, Hsp90 engages in a dynamic process characterized by numerous protein-protein interactions (PPIs) with various co-chaperones. Prominent co-chaperones include the Hsp70/Hsp90 organizing protein (Hop), Cell Division Cycle 37 (cdc37), and ATPase homologue (Aha1)), all of which are associated with Hsp90 at different stages of the chaperone cycle (a summary can be found in Figure 8). At the onset of the Hsp90 chaperone cycle, client proteins are loaded onto Hsp90 by co-chaperones. Hop facilitates this transfer by use its tetracopeptide (TPR) domain to interact with the EEVD motif of Hsp90 located on the CTD (Backe et al., 2022; Lorenz et al., 2014). This step is particularly critical when transferring clients, such as glucocorticoid receptors, from a complex with Hsp70 to Hsp90. Conversely, protein kinases and certain other client proteins depend on cell division cycle 37 (Cdc37/p50) for their recruitment to Hsp90 (Calderwood, 2015). Aha1 is thought to bind to the NTD and MD to enhance the otherwise low basal ATPase activity of Hsp90 (Calderwood, 2015; Retzlaff et al., 2010). Meanwhile, the NTD-binding protein p23 tends to inhibit ATPase activity, thereby prolonging the interaction of Hsp90 with its clients (Cox & Johnson, 2011; Sullivan et al., 2002). The inhibition of Hsp90 ATPase activity by p23 serve as a notable regulatory factor in the chaperone cycle, with p23 being involved in the late complex during the final stages of protein maturation.

A comprehensive list- maintained by Dider Picard- of mainly mammalian co-chaperones and clients of Hsp90 can be found on: http://www.picard.ch/downloads/downloads.htm. The list highlights clients that rely on Hsp90 for folding, stability, and activity, as well as co-chaperones that regulate Hsp90 and client protein functions. Given the essential role of co-chaperones in fine-tuning the activity of Hsp90 for protein folding, recent developments suggest that disrupting the interactions between co-chaperones and Hsp90 could serve as an alternative and specific method to regulate the chaperone cycle without inhibiting ATPase activity. Some research has been conducted in this area focusing on disrupting the Hsp90-cdc37 and Hsp90-Hop interaction as potential strategies in cancer (Chen et al., 2018; Dernovšek, Gradišek, et al., 2024; Okpara et al., 2024; Wang et al., 2019).

In the case of *P. falciparum*, research indicates that the role of *P. falciparum* Hsp90 extends beyond maintaining cellular homeostasis. Hsp90 and other heat shock proteins are implicated in the development and pathogenesis of the organism (as reviewed by (Shonhai et al., 2011)). Malarial parasites must adapt to harsh temperatures and various environmental stresses throughout their life cycles within their hosts, necessitating a robust molecular chaperone response (Shonhai et al., 2011). However, the co-chaperone network associated with PfHsp90 has yet to be extensively studied; it is important to note that the co-chaperone network in *P. falciparum* differs from that of its human host (as reviewed by (Dutta et al., 2022)). There remains a need for further data regarding the inhibition of the PfHsp90 and co-chaperone complex. Notably, phenylthynesulfonamide has been shown to disrupt the functional cooperation between PfHop and PfHsp70–1 *in vitro* (Muthelo et al., 2022).

## Rational design strategies targeting PfHsp90

Rational drug design (RDD) is a process in which drugs are developed with knowledge of a target molecule’s structure and function (Batool et al., 2019). The process involves using information about the molecular target, such as its 3D structure, to design a molecule that interacts specifically with that target to produce a therapeutic effect (Kholodenko, 2000). RDD aims to create more effective and specific drugs with fewer side effects than traditional phenotypic screening approaches (Mahapatra & Karuppasamy, 2022). The target’s molecular structure is used to predict how a drug molecule will interact with it, and the design process can involve computer simulations, molecular modelling, and synthesizing new compounds to test their efficacy (Mahapatra & Karuppasamy, 2022).

RDD has emerged as a central theme in several studies aimed at developing small molecule inhibitors targeting PfHsp90, contributing to the search for new antimalarial therapies. Research conducted by Wang et al. (2014 and 2016), Everson et al. (2021), and Mafethe et al. (2023) offered valuable insights into the effective application of RDD for generating selective inhibitors of PfHsp90. Wang et al. (2014) established the groundwork for employing RDD focused on PfHsp90 by examining the structural differences between the ATP-binding pockets of PfHsp90 and its human counterpart. Through computational docking studies, unique amino acid residues within the ATP-binding site of PfHsp90 that could be targeted for selective inhibition were described. Leveraging this information, the investigators designed a series of compounds that specifically interacted with these distinct features, resulting in inhibitors that preferentially bound to PfHsp90 while exhibiting minimal activity against human Hsp90. This study demonstrated the potential for designing selective PfHsp90 inhibitors based on structural insights. In a subsequent study, Wang et al. (2016) further refined their rational drug design strategy by integrating advanced computational techniques, including molecular dynamics simulations and cheminformatics analyses. These approaches facilitated a deeper understanding of the dynamic interactions between inhibitors and PfHsp90, particularly in terms of how these compounds could induce conformational changes that enhance selectivity and binding affinity. The research identified a second-generation series of inhibitors characterized by improved potency and selectivity, underscoring the evolution of rational design strategies aimed at optimizing lead compounds.

On the other hand, Everson et al., (2021) study took a slightly different approach by integrating structure-based drug design with virtual screening. Initially, they employed high-throughput screening to identify lead compounds with activity against *P. falciparum*. The subsequent rational design was informed by the crystal structure of PfHsp90 in complex with initial hits, revealing fundamental interactions and potential areas for optimization. This iterative process of phenotypic screening, followed by rational optimization based on structural analysis, facilitated the development of highly selective inhibitors with potent antimalarial activity, illustrating the power of combining phenotypic and structure-based approaches.

The recent study by Mafethe et al., (2023) represents a culmination of advancements in rational drug design targeting PfHsp90. This research used an integrated approach that combined virtual library screening and detailed structure activity relationship (SAR) studies to design novel PfHsp90 inhibitors. The team could predict and generate new inhibitors with high selectivity and potency against PfHsp90 by analyzing large datasets of compound structures and their biological activities. Furthermore, the study emphasized the importance of understanding the physicochemical properties that influence drug-likeness and pharmacokinetics, leading to the identification of lead compounds with favorable profiles for clinical development. These studies illustrate the evolution and sophistication of rational drug design strategies targeting PfHsp90. From initial structure-based designs to integrating computational, phenotypic, and machine learning approaches, the research highlights a concerted effort to develop selective PfHsp90 inhibitors as potential antimalarial agents. By focusing on the unique attributes of PfHsp90 and continually refining design strategies, these studies contribute significantly to the field of antimalarial drug discovery.

## Artificial intelligence

The application of artificial intelligence (AI) in drug discovery represents a transformative shift in our approach to developing new treatments for malaria. AI methodologies, encompassing machine learning (ML) and deep learning (DL), offer unprecedented opportunities to accelerate the identification and optimization of PfHsp90 inhibitors, while also presenting a set of challenges unique to this high-tech approach (Xia et al., 2024). Recent years have seen a significant increase in the application of AI in drug discovery, with emphasis on predicting the binding affinity of potential inhibitors, streamlining the drug design process, and identify novel drug-like molecules with high specificity and potency (Han et al., 2023; Vijayan et al., 2022; Vora et al., 2023). One notable advancement in developing AI-driven predictive models is accurately forecasting potential inhibitors’ pharmacokinetic and toxicological profiles, addressing a critical step in the early stages of drug development (Tran et al., 2023).

The design of selective PfHsp90 inhibitors that minimize off-target effects and toxicity is another area where AI has made substantial contributions (Visan & Negut, 2024). By integrating structure-based drug design (SBDD) with ML algorithms, researchers have been able to predict the selectivity of compounds more accurately (Batool et al., 2019). While AI offers a promising avenue for accelerating the discovery and development of PfHsp90 inhibitors, several challenges remain. The accuracy of AI predictions heavily depends on the quality and quantity of the underlying data (Blanco-González et al., 2023).

In summary, integrating AI into the drug discovery process targeting PfHsp90 is rapidly evolving, offering new possibilities to overcome traditional barriers. By leveraging the power of AI to predict, design, and optimize PfHsp90 inhibitors, researchers have made significant strides toward the development of novel antimalarial therapies. However, to fully realize the potential of AI in this domain, ongoing efforts to improve model transparency, accuracy, and the integration of experimental validation are essential.

## Conclusion

The exploration of PfHsp90 as a target for malaria treatment represents a promising yet challenging frontier in the fight against this enduring global health menace. The comprehensive review of the possibilities and difficulties associated with PfHsp90 inhibition underscores the potential of this target to address the urgent need for novel antimalarial therapies, particularly in the face of rising drug resistance. The unique role of Hsp90 in the *P. falciparum* parasite biology and the successful identification of inhibitors that can disrupt its function highlights a viable path toward developing effective treatments. However, the journey from concept to clinical application is fraught with hurdles, including achieving selective toxicity, overcoming pharmacokinetic limitations, and preventing the emergence of resistance. The current landscape of PfHsp90 research indicates a burgeoning interest in overcoming these challenges through innovative drug design, rigorous pre-clinical testing, and the integration of computational tools to predict drug behavior. Moreover, the potential synergy of PfHsp90 inhibitors with existing antimalarial agents offers a compelling strategy to enhance therapeutic efficacy and mitigate resistance.

As we progress, research in this area must continue to be dynamic, multidisciplinary, and collaborative, encompassing molecular biology, pharmacology, medicinal chemistry, and computational modeling. Equally important is considering access and affordability to new treatments in malaria-endemic regions, underscoring the need for global cooperation and investment. The journey of translating PfHsp90 inhibition from a theoretical concept into a practical antimalarial strategy is symbolic of the broader challenges and opportunities in drug discovery and development. Embracing the complexity of this task while being guided by the principles of innovation, collaboration, and equity will be crucial in our collective efforts to devise practical solutions against malaria. Through persistent research and development, PfHsp90 inhibitors have the potential to play a pivotal role in the next generation of malaria treatments, contributing significantly to the global endeavor to eradicate this disease.

## Figures and Tables

**Table 1:** Target Candidate Profiles (TCP) that describe the required modes of action of new potential antimalarial compounds (Burrows et al., 2017; Forte *et al.,* 2021; MMV, 2024).

TCP	Goal	Definition
TCP-1	Treatment of severe and uncomplicated malaria, as well as offering protection to vulnerable population groups.	Compounds must be active against the asexual blood stage of the *Plasmodium* life cycle and active against all resistant strains.
TCP-3	Anti-relapse (treatment of recurrent malaria)	Compounds must be active against liver-stage hypnozoites.
TCP-4	Prophylaxis (for migrating population groups or preventing outbreaks)	Compounds must be active against liver stages and ideally protect for at least a month
TCP-5	Transmission blockers (prevention strategies that include the treatment of asymptomatic infection)	Compounds active against parasite gametocytes
TCP-6	Transmission blockers	Compounds that block transmission by targeting the mosquito vector (insecticides)

**Table 2:** Antimalarial drug candidates currently in clinical trials adapted from (Umumararungu et al., 2023).

Drug name	Chemical class	Phase	Target and mechanism of action
ACT-451840	Phenylalanine	I	The mechanism of action is not yet known (Le Bihan et al., 2016).
CDRI-97/78	1,2,4-Trioxane	I	Inhibits phospholipid metabolism of *P. falciparum* blood-stage multiplication (Shafiq et al., 2014).
Ganaplacide	Imidazolopiperazine	I	Inhibits protein trafficking of *Plasmodium* spp. and blocks the establishment of new permeation pathways (Ogutu et al., 2023).
MMV048	Aminopyridine	II	Inhibits phosphatidylinositol 4-kinase (PI4K) (Paquet et al., 2017).
P218	2,4-Diamino-pyrimidine	I	Inhibits *P. falciparum* dihydrofolate reductase (*Pf*DHFR) (Yuthavong et al., 2012).
DM1157	4-Aminoquinoline	I	Inhibits *β*-hemozoin formation (Fong & Wright, 2013)
MMV533	Acylguanidine	I	The mechanism is hypothesized to be inhibition of lipid storage and/or vesicular trafficking pathways (Murithi et al., 2021).
INE963	5-Aryl-2-amino-imidazothiadiazole	I	The mechanism of action is unknown (Taft et al., 2022)
GSK701	Pyrrolidinamide	I	Inhibits *P. falciparum* acyl-CoA synthetase 10/11 (Bopp et al., 2023).
ZY-19489	Triaminopyrimidine	I	Inhibits vacuolar ATP synthase (Barber et al., 2022)
SJ733	Dihydroisoquinolone	II	Disrupts the ATP4 function of *P. falciparum* (Gaur et al., 2022)
AQ-13	4-Aminoquinoline	II	The mechanism of action has not yet been evidenced (Mengue et al., 2019).
Cipargamin	Spiroindolone	II	Blocks mosquitos’smission and inhibits *Pf*ATP4 (Goldgof et al., 2016).

**Table 3:** The binding affinity and inhibitory activity of Hsp90 inhibitors against *P. falciparum* parasites and mammalian cells.

Compound	PfHsp90 K_d_ (μM)	Antiplasmodial IC_50_ (μM)	Mammalian IC_50_ (μM)	Selectivity Index	References
GDA	1.05	0.02	0.04	1.6	Pallavi et al., 2010
17-AAG	4.52	0.16	0.09	0.6	Murillo-Solano et al., 2017
17-DMAG	0.008	0.12	0.3	2.5	Murillo-Solano et al., 2017
PU-H71	70.8	0.11	3.92	35.3	Shahinas et al., 2013
Harmine	40.0	0.05	ND	ND	Shahinas et al., 2012
IMA	19.4	1.42 **	>50	>35	Nndwammbi et al., 2024, Nyaba *et al.,* 2018
UAA	8.16	4.16**	>50	>13	Nndwammbi et al., 2024, Simelane *et al.,* 2013
FM2	13.5	0.14	>50	>357	Mafethe et al., 2023
FM4	2.45	0.92	6.98	>54	Mafethe et al., 2023
FM6	0.21	1.76	12.82	>28	Mafethe et al., 2023

a ND = not determined

** Note the AUU and IMA were reported in μg/mol which were converted to μM concentrations for uniformity of the data.

**Table 4:** Binding affinities of GA and derivatives towards PfGRP94 and HsHsp90

Compound	PfGRP94 K_d_ (μM)	HsHsp90 K_d_ (μM)	Selectivity Index	References
GDA	1530	1110	0.73	Murrilo-Solano et al., 2017
17-AAG	28.5	0.09	0.003	Murrilo-Solano et al., 2017
17-DMAG	0.02	0.16	6.86	Murrilo-Solano et al., 2017
PU-H71	6.43	0.27	0.04	Murrilo-Solano et al., 2017
